# Early Neurodevelopmental Findings Predict School Age Cognitive Abilities in Duchenne Muscular Dystrophy: A Longitudinal Study

**DOI:** 10.1371/journal.pone.0133214

**Published:** 2015-08-14

**Authors:** Daniela Chieffo, Claudia Brogna, Angela Berardinelli, Grazia D’Angelo, Maria Mallardi, Adele D’Amico, Paolo Alfieri, Eugenio Mercuri, Marika Pane

**Affiliations:** 1 Department of Paediatric Neurology, Catholic University, Rome, Italy; 2 Child Neurology and Psychiatry Unit, “C. Mondino” Foundation, Pavia, Italy; 3 IRCCS Eugenio Medea, Bosisio Parini, Italy; 4 Department of Neurosciences, Unit of Neuromuscular and Neurodegenerative Disorders, Bambino Gesù Children’s Hospital, Rome, Italy; 5 Child and Adolescence Neuropsychiatry Unit, Department of Neuroscience Bambino Gesù Children’s Hospital, Rome, Italy; The Hospital for Sick Children, CANADA

## Abstract

**Objective:**

Neurodevelopmental and cognitive difficulties are known to occur frequently in boys with Duchenne muscular dystrophy but so far none of the published studies have reported both early neurodevelopmental assessments and cognitive tests in the same cohort. The aim of the present longitudinal study was to establish the correlation between early neurodevelopmental assessments performed in preschool boys and the cognitive scales performed at school age or later.

**Methods:**

We performed cognitive tests at school age (mean age 5.7 year ±1.7 SD) (69 months+19 SD) in a cohort of Duchenne boys, previously assessed using the Griffiths scales before the age of 4 years (mean age when the Griffiths scales were performed 30 months ±8.9 SD).

**Results:**

The range of total Developmental quotients on the Griffiths ranged between 56 and 116 (mean 89 ± 15.6 SD). The total Intelligence Quotients on the Wechsler scales ranged between 35 and 119 (mean 87 ± 17.2 SD). There was a significant correlation between the findings on the two scales. P = <0.0001. When we subdivided the cohort according to site of mutations, there was a difference between boys with mutations upstream exon 44 and those with mutations in exon 44–45 affecting Dp140 on both Developmental and Intelligence Quotient (*p 0*.*01* and *p 0*,*003* respectively).

**Conclusions:**

Our results confirm that Duchenne boys tend to slightly underperform on both neurodevelopmental and cognitive assessments. Early neurodevelopmental findings correlated with the cognitive results obtained at school age with a clear concordance between subscales exploring similar domains on the two scales.

## Introduction

Duchenne muscular dystrophy (DMD) is a hereditary, X-linked, progressive, muscular disorder affecting about 1 in 3500 boys [1,2]. Cognitive impairment is present in approximately one third of the patients [3, 4, 5, 6, 7] with reported mean Intelligence Quotients (IQ) scores approximately 1.0 to 1.5 standard deviations below the mean for age matched typically developing children [8, 9, 10, 11, 12, 13, 14]. A meta-analysis performed [15] in 721 patients with DMD indicated that the overall mean intelligence quotient was 82 (approximately 1 SD below the population mean). Longitudinal studies have suggested that the IQ tend to remain stable with increasing age [11, 16, 17]. Most of these studies however have assessed boys and young adults at school age or later. Less has been reported in younger, preschool children. A few recent studies using neurodevelopmental assessments such as the Griffiths [18] and the Bayleys [19] scales in relatively large cohorts of young children have reported that early neurodevelopmental abnormalities can already be detected in preschool children. In our previous study [18], using the Griffiths scales the mean Developmental Quotient (DQ) was 87, approximately one standard deviation below the mean, in keeping with data reported in older children assessed on cognitive scales [6, 20, 21]. So far none of the published studies have performed both early neurodevelopmental assessments and cognitive tests in the same cohort.

The aim of the present longitudinal study was to establish the correlation between early neurodevelopmental assessments and the cognitive scales in a cohort of preschool young DMD boys followed until school age.

## Subjects and Methods

The study is a longitudinal multicentric study involving four tertiary neuromuscular centers in Italy (Catholic University and Bambino Gesù Hospital Rome; IRCCS “C. Mondino” Foundation, Pavia; and IRCCS E. Medea, Bosisio Parini). Children were included if they had a genetically proven DMD diagnosis or absence of dystrophin on muscle biopsy. The genetic analysis, searching for exons, deletions, and duplications was performed using Multiplex ligation-dependent probe amplification (MLPA). In patients with deletions and duplications, all 79 exons and the adjacent introns were analyzed through Polymerase Chain Reaction (PCR) amplification and direct sequencing. Mutations were named according to the Leiden Muscular Dystrophy database (http://www.dmd.nl/) using the nomenclature system published in 2000 in Human Mutation. As part of our clinical practice, patients are routinely assessed with age-appropriate neurodevelopmental and, at school age, with cognitive scales. In this study we only included patients who were first seen before the age of 4 years, excluding those who came to our observation after the age of 4 years for whom earlier tests were not available. The study was approved by the Ethics Committee of all centers.

### Neurodevelopmental and cognitive assessment

Neurodevelopmental outcome was evaluated by chartered psychologists and pediatric neurologists using the Griffiths Scale of Mental Development. The scale includes five subscales (locomotor, personal-social, hearing and speech, hand and eye co-ordination and performance) and, for the children older than two, an additional subscale assessing practical reasoning [22, 23]. One of the advantages of this test is that it provides not only a global developmental quotient but also subquotients for the individual five subscales: Locomotor (A), Personal Social (B), Hearing and speech (C), Eye hand Coordination (D) and Performance (E). The scale is routinely administered to all the boys who come to our observation before the age of 4 years.

All the children were reassessed at school age using the Wechsler scales. More specifically, we used the Wechsler Intelligence Primary and Preschool Intelligence Third Revision scale (WPPSI III) [24] for boys between the age of 4 years and 6 years and the Wechsler Intelligence School Children third Revision (WISC III) [25, 26] for those older than 6 years. These scales provide a full Scale Intelligence Quotient (FSIQ), a verbal Intelligence Quotient (VIQ) and a performance Intelligence Quotient (PIQ). They include 12 subtests (six verbal and six performance) which investigate cognitive functions, such as verbal and arithmetical reasoning, judgement in practical situations, visual analysis, perceptive organization as well as visual motor and visual spatial planning.

### Statistical analysis

Spearman rank correlation was used to test correlations between Griffith DQ and Wechsler FSIQ.

In order to reduce the possible effect of the locomotor scale on the full DQ the results were also analyzed taking out the locomotor scale from the total DQ. Similarly, in order to correlate verbal and performance IQ on the Wechsler scales with specific subscales of the Griffiths we performed further analysis comparing verbal IQ with hearing and speech subscale on the Griffiths and performance IQ with eye hand coordination and performance subscales on the Griffiths.

Our cohort was also subdivided into three subgroups:1) boys with mutations upstream or in the exon 44, known to affect expression of the full length dystrophin isoforms (n = 17); 2) boys with mutations between exon 44 and 55, predicted to additionally affect Dp140 expression (n = 19); 3) boys with mutations downstream of exon 62, known to affect all the brain isoforms of dystrophin, including the lower molecular weight Dp71 isoform (n = 2).

As there were only 2 boys with mutations downstream of exon 62, the analysis was performed on the first two subgroups only.

The effects of age class, site and type of mutation as defined above on neurodevelopmental assessment performed by the Griffiths Scale scores (DQ) and the Wechsler scales as continuous variables were evaluated by an Analysis of Variance (ANOVA) model. The level of significance was set at 0.05.

## Results

All the families of 41 patients who were asked to participate to the study consented to enrolment.

Details of their mutations were available in 38 out of the 41.

### Neurodevelopmental assessment

The mean age when the Griffiths scales were performed was 30 months ±8.9 SD (2.5 years ±0.7). The range of total DQ ranged between 56 and 116 (mean 89 ± 15.6 SD).

Details of the tests are shown in Table 1.

**Table 1:** Mean and SD of specific Neurodevelopmental and Wechsler Subquotients.

	Griffiths scales					Wechsler	
	A	B	C	D	E	VIQ	PIQ
IQ Range	55–120	58–115	55–125	45–120	50–122	39–112	46–131
Mean and SD	80±17.6	93 ±16.1	91±20.8	86±18.2	93±18.2	87±14.7	90±19.6

### Cognitive assessment

The mean age when the Wechsler scales were performed was 69 months+19 SD (5.7 years ±1.7 SD).

Total IQ ranged between 35 and 119 (mean 87 ± 17.2 SD).

Details of the tests are shown in Table 1.

### Correlation between early neurodevelopmental and school age cognitive findings

A significant correlation (<0.0001) was found between the total IQ on the Wechsler scales and the full DQ also when the locomotor scale was excluded.

When we analyzed the correlation between Wechsler subquotients and specific Griffiths subscales, a significant correlation was observed between VIQ and hearing and speech Griffiths subscale (0.0032).

The correlation was also significant between performance IQ and performance (<0.0001) and eye and hand coordination (<0.0001) subscales on the Griffiths (Table 2).

**Table 2:** Correlation between specific Neurodevelopmental and Wechsler subquotients.

	A group (n 17)	B group (n 19)	C group (n 2)
**DQ *Range***	73–116	56–106	57–62
***mean and SD***	97±13.7	87 ±12.2	59.5±3.5
**FIQ *Range***	81–126	57–101	63–75
***mean and SD***	95±13.4	82± 12.7	69± 8.4

When we subdivided the cohort according to site of mutations, there was a difference between boys with mutations upstream exon 44 and those with mutations in exon 44–45 affecting Dp140 (Table 3) on both DQ and IQ (*p 0*.*01* for DQ and *p 0*,*003* for FSIQ).

**Table 3:** DQ and FSIQ range, mean and SD in A, B, C group. **A group**: Patients with mutations upstream exon 44 (n 17), **B group**: Patients with mutations in exon 44–55 predicted to affect Dp140 (n19), **C group**: Patients with mutations downstream of exon 62, known to affect all the brain isoforms of dystrophin, including the lower molecular weight Dp71 isoform (n = 2).

	A group (n = 17)	B group (n = 19)	C group (n = 2)
**DQ *Range***	73–116,	56–106	57–62
***mean and SD***	97±13.7	87 ±12.2	59.5±3.5
**FIQ *Range***	81–126	57–101	63–75
***mean and SD***	95±13.4	82± 12.7	69± 8.4

## Discussion

We recently reported early neurodevelopmental findings in a cohort of DMD boys assessed before the age of 4 years using the Griffiths scales [18]. The mean DQ was 87, approximately one standard deviation below the mean. Not surprisingly, the lowest scores were related to the locomotor scale (subscale A) but low DQ were also found in hearing and speech (subscale C) and other subscales such as eye and hand coordination and performance suggesting early difficulties also on more cognitive aspects. These results appeared to be in keeping to those found in older boys assessed using cognitive scales [8,16]. As the neurodevelopmental scales used in preschool children and the cognitive scales used in the older boys however have different constructs and none of the studies reported so far have used the two assessments longitudinally, it was difficult to ascertain whether the early neurodevelopmental findings were related to the school age cognitive difficulties found in other papers.

In the present study cognitive we performed tests at school age in a cohort of DMD boys, previously assessed using the Griffiths scales before the age of 4 years. Mean scores below 1 SD were found both on neurodevelopmental scales and cognitive assessments. There was a significant correlation between the findings on the two scales, suggesting that even before school age, the level of cognitive functioning in DMD remains quite stable, as observed in older patients.

Not surprisingly there was a strong correlation between verbal IQ and hearing and speech subscale on the Griffiths. Language delay is one of the possible presenting signs of DMD [3, 10, 27, 28] and early difficulties on the verbal subscale, when present, appear to persist at school age, as also demonstrated by several previous studies showing that verbal IQ is generally more affected than the performance IQ.

Similarly the correlation between performance IQ and eye and hand coordination and performance subscales confirms that early difficulties in these aspects can be present even at a younger age. They appear to be as part of a more global pattern of cognitive impairment rather than related to upper limb weakness that in young children is not present [29].

The correlation between the full score IQ on the Wechsler scales and the total DQ that also includes the locomotor subscales raises another important point. As boys affected by DMD generally show early signs of weakness, with inability to run fast or jump, we were concerned that this may affect the scores of locomotor scale with a direct correlation with the total DQ. When we analysed the results however we found that the correlation between full score IQ on the Wechsler scales and the total DQ was significant (<0.0001) also when the locomotor scale was excluded.

Furthermore, as reported by us and others [3,4], not all the abnormalities on the locomotor scale are strictly related to early weakness. A number of motor abilities, such as standing on one leg or walking up and down stairs, are often achieved with a delay, and therefore are more likely to be the expression of the involvement of the brain dystrophin isoforms that is responsible for a global neurodevelopmental delay rather than to weakness that would not improve with increasing age [4]].

The concordance between the findings on early neurodevelopmental and cognitive scales was further confirmed by the analysis of the subgroups according to site of mutation. On both scales the range of findings was similar when the boys were subdivided according to whether mutations were upstream or involving/downstream exon 44. Patients with mutations upstream exon 44 that do not involve the brain isoforms had overall better results than those with mutations downstream exon 44 both on the Griffiths and the Wechsler scales suggesting that the effect of site of mutation and the involvement of dystrophin isoforms not only is already obvious in the first years but remain also stable over time.

In conclusion, our results confirm that DMD boys tend to slightly underperform on both neurodevelopmental and cognitive assessments with a relatively wide range of quotients. Early neurodevelopmental findings correlate with the cognitive results obtained at school age with a clear concordance between subscales exploring similar domains on the two scales. These results suggest that early neurodevelopmental assessments should be routinely performed in DMD boys as they can allow early detection of early difficulties and help to plan early intervention. The site of mutation can provide further help in identifying boys at risk of cognitive impairment.
